# Kinetic Analysis of Water Fitness Exercises: Contributions for Strength Development

**DOI:** 10.3390/ijerph16193784

**Published:** 2019-10-08

**Authors:** Catarina C. Santos, Luís M. Rama, Daniel A. Marinho, Tiago M. Barbosa, Mário J. Costa

**Affiliations:** 1 Faculty of Sports Science and Physical Education, University of Coimbra, 3040-256 Coimbra, Portugal; catarina.costa.santos@ubi.pt (C.C.S.);; 2 Research Unit for Sport and Physical Activity, CIDAF, 3040-256 Coimbra, Portugal; 3 Department of Sport Sciences, University of Beira Interior, 6201-001 Covilhã, Portugal; 4 Research Center in Sport, Health and Human Development, CIDESD, 5001-801 Vila Real, Portugal; 5 Department of Physical Education & Sports Sciences, Nanyang Technological University, Singapore 637616, Singapore; 6 Department of Sports Sciences, Polytechnic Institute of Bragança, 5300-252 Bragança, Portugal; 7 Department of Sports Sciences, Polytechnic Institute of Guarda, 6300-559 Guarda, Portugal

**Keywords:** water exercise, propulsive force, isometric force, asymmetries, cadence

## Abstract

The evaluation of propulsive forces in water allows the selection of the most appropriate strategies to develop strength during water fitness sessions. The aim of this study was threefold: (i) to analyze the rate of force production; (ii) to analyze the rate of force variation; and (iii) to compare limbs’ symmetry in two water fitness exercises. Twenty-two young health subjects (age: 21.23 ± 1.51 years old, body mass: 67.04 ± 9.31 kg, and height: 166.36 ± 8.01 cm) performed incremental protocols of horizontal adduction (HA) and rocking horse (RH_add_), from 105 until 150 b·min^−1^. Data acquisition required an isokinetic dynamometer and a differential pressure system that allowed the assessment of (a) isometric peak force of dominant upper limb (IsometricF_D_); (b) propulsive peak force of dominant upper limb (PropulsiveF_D_); and (c) propulsive peak force of nondominant upper limb (PropulsiveF_ND_). Significant differences were found in the rate of force production (RateF_D_) between the majority cadences in both exercises. The RateF_D_ reached ~68% of the force in dry-land conditions, and lower cadences promoted a higher rate of force variation (ΔForce). Most actions were asymmetric, except for the HA at 135 b·min^−1^. In conclusion, the musical cadence of 135 b·min^−1^ seems to elicit a desired rate of force production with a symmetric motion in both exercises.

## 1. Introduction

Aquatic activities related to health and well-being promotion increased remarkably in popularity and adherence in the past decades. There is a wide variety of water programs focusing on fitness [1], performance [2], rehabilitation [3], and therapy [4]. The increasing interest is attributed to the potential benefits of water programs, as reported in the literature [5]. Other potential benefits mentioned include (i) the reduced effect of body weight; (ii) the reduced impact in specific joints (e.g., a decreased ground reaction force); (iii) the reduced muscle pain; (iv) an improved blood flow due to hydrostatic pressure; (v) a three-dimensional body motion and; and (vi) higher social development and commitment. Previous reports focused on acute and chronic physiological adaptations [6]. However, there is a lack of knowledge on the biomechanical changes, such as the impact on the kinetics.

Measurements of water forces were made through the years. However, pressure sensors seem the most suitable instrument, allowing free motion during testing without constraints. Those differential pressure sensors were validated [7] and allow for the measurement of propulsive forces in an ecological validity environment. There is little data on this topic, and the existing body of knowledge is based on evidence gathered in competitive swimmers and patients [8,9]. Prins, Hartung, Merritt, Blancq, and Goobert [8] noted, in clinical population (poliomyelitis disability), values near to 45 and 60 N for the right and left hand, respectively, during horizontal arms adduction at maximum velocity. This evaluation of propulsive forces in water allows the selection of the most appropriate strategies to develop strength during water fitness sessions. For instance, water fitness professionals can know at which rate of the maximal force their clients are practicing on water.

Human bodies are expected to be asymmetrical in nature, as their force production ability is. Based on this reasoning, force data acquisition may provide new insights into the critical aspects of motion, such as muscular imbalances [10,11]. Muscle imbalances can elapse from asymmetric actions while exercising and increase the susceptibility to a chronic injury. The persistence in asymmetric patterns can deteriorate the current status of a given joint, impairing, in some cases, daily life activities. Thus, is important to dissect how kinetic behavior or coordination changes in water fitness sessions, considering different sort of stimuli.

In the past, Robinson, Herzog, and Nigg [12] designed and proposed a Symmetric Index (SI) to assert the asymmetries that result from ground reaction forces during the gait cycle. Nowadays, in time-based sports, such as running or swimming, the SI is used to demonstrate asymmetric patterns and their relationship with acute or chronic injury [13,14]. While testing swimmers, Morouço et al. [15] showed that the majority of the subjects (66.7%) had an asymmetrical force production. To the best of our knowledge, this kind of study was never done in water fitness exercises. A feasible way is to understand the role of propulsive forces and symmetry at different music cadences. This approach will help water fitness professionals to prescribe and define the most appropriate music cadences, to obtain a desirable force production and coordination in each stage of the session. Moreover, it will allow us to determine the real rate of force production in water as compared to maximal strength obtained from dry-land testing.

The aim of this study was threefold: (i) to analyze the rate of force production of the dominant upper limb in water compared to dry-land data; (ii) to analyze the rate of force variation during the incremental protocol; and (iii) to assess symmetry in horizontal adduction and rocking horse at different cadences. It was hypothesized that the rate of force production would increase to follow the musical cadence and that values could be above 50%, considering dry-land data. Likewise, the increase of propulsive peak force at higher cadences would promote an asymmetrical movement.

## 2. Materials and Methods

### 2.1. Participants

Twenty-two young health subjects, nine women and thirteen men (age: 21.23 ± 1.51 years old, body mass: 67.04 ± 9.31 kg, height: 166.36 ± 8.01 cm), volunteered to participate in this study. The following inclusion criteria were considered: (i) being clinically healthy and physically active; (ii) having at least one year of experience in water fitness programs; (iii) being nonpregnant; and (iv) not having muscle-skeletal or neurologic injuries, conditions, or syndromes diagnosed in the past six months. All participants were informed of the benefits and experimental risks prior to giving their written informed consent for the participation. All procedures were in accordance with the Helsinki Declaration in respect to human research, and they had local ethics board approval.

### 2.2. Design and Procedures

**Figure 1:**
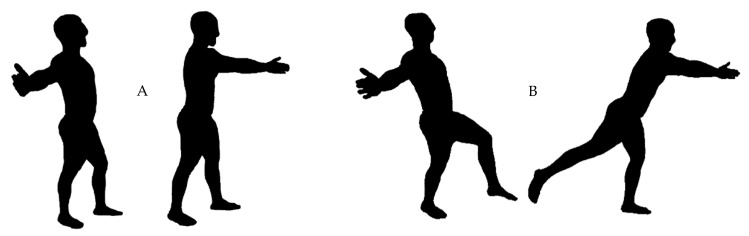
The basic water fitness exercises, “horizontal adduction” (**A**) and “rocking horse” (**B**).

In-water data collection was held in a 25 m indoor pool (12.5 m width and maximal depth of 1.80 m), with a mean water temperature of 29 °C. Participants were randomly assigned to perform in different days the following water fitness exercises (Figure 1): (A) horizontal arms adduction (HA) and (B) rocking horse with horizontal arms adduction (RH_Add_). The HA is characterized as maintaining a static trunk with lower limbs fixed to the ground [16] when performing the upper-limbs action. During the motion of the arms, full extension is required, without any restriction in the range of motion of shoulders at abduction. Both hands are positioned at a 90° angle, considering the water surface. In RH_Add_, the upper limbs have the same pattern of motion as in HA. The lower limbs’ actions show a continuous and simultaneous motion, with horizontal arms adduction and abduction [17]. In every cycle between leaps, there was a knee flexion when the participants’ performed arms abduction, while the opposite leg did a hyperextension. The level of water surface was set at near xiphoid process, as recommended by Barbosa, Garrido, and Bragada [5].

All selected exercises are prescribed as regular basic exercises in water fitness programs. Each exercise was performed over an incremental protocol, with 4 music cadences, starting at 105 beats per minute (b·min^−1^) and increasing every 30 seconds by 15 b·min^−1^, up to 150 b·min^−1^. The music cadence was controlled by a metronome (Korg, MA-30, Tokyo, Japan) that was plugged in to a sound system, and both exercises were performed at “water tempo”, characterized by the countdown of only one beat in every two beats [18], permitting the music to be synchronized with the specific movement. Verbal and visual feedback was given during every cadence. The test was concluded when the participant reduced the range of motion and failed the set cadence or when the participant completed the 30 seconds at each cadence.

Dry-land data were conducted to analyze isometric force production, using an isokinetic dynamometer (Biodex Multi-Joint System 3 Pro, Shirley, NY, USA). Two groups performed a 3-minute warm-up on a stable upper-body ergometer (Monark 891E, Vansbro, Sweden). Cadence was set between 70 and 80 rpm. A 2-repetition trial was conducted before each test for familiarization purposes [19]. Immediately, a 3-repetition protocol with dominant the upper limb in adduction at 45° was performed during 6 s of maximal isometric force and the 15 s interval between sets [20]. Isometric peak force of dominant member (IsometricF_D_) was considered at the best repetition and expressed in Newton (N).

### 2.3. Measures

Propulsive forces were assessed by a hydrodynamic measurement system previously validated [7] with 0.2% of measurement error. The system is composed of two independent sensors that are positioned between the phalanges of the middle and ring fingers of both hands and allow assess to peak force of dominant (PropulsiveF_D_) and nondominant (PropulsiveF_ND_) upper limbs in Newton (N). A signal-processor (AcqKnowledge v.3.7.3, Biopac Systems, Santa Barbara, CA, USA) was used to export data with a 5 Hz cutoff low-pass 4th order Butterworth filter upon residual analysis. The first positive and negative peak (one cycle) were discarded. Symmetric Index (SI, %) was estimated as proposed by Robinson, Herzog, and Nigg [12]:

(1) $$\mathrm{SI}\;\left(\%\right)=\frac{2\left(x_{d}-x_{\mathrm{nd}}\right)}{\left(x_{d}+x_{\mathrm{nd}}\right)}\times 100$$

where $x_{d}$ represents the force produced by the dominant upper limb, and $x_{\mathrm{nd}}$ represents the force produced by the nondominant upper limb.

Symmetry data was interpreted as suggested by the same authors, where if SI = 0%, there was perfect symmetry; if 0% ˃ SI < 10%, there was symmetric motion; and if SI ≥ 10%, there was asymmetric motion. The rate of force production for the dominant upper limb (RateF_D_) was considered as follows: (100 × PropulsiveF_D_)/(IsometricF_D_).

### 2.4. Statistical Analysis

Exploratory data analysis was used to identify potential outliers. The Shapiro–Wilk test was used to confirm the normality of distribution (*p* > 0.05). Descriptive statistics (mean, standard deviation, and 95% of confidence interval) are reported. The relationship between water and dry-land conditions was assessed by stepwise regression analysis. The Friedman test was conducted to compare differences between cadences. Additionally, effect size (ES) was calculated based on Cohen’s *d* [21], to assess the magnitude of the mean differences between cadences and interpreted, according to author’s recommendation: (i) small (*d* ≥ 0.20); (ii) moderate (*d* ≥ 0.50); and (iii) large (*d* ≥ 0.80).

## 3. Results

**Figure 2:**
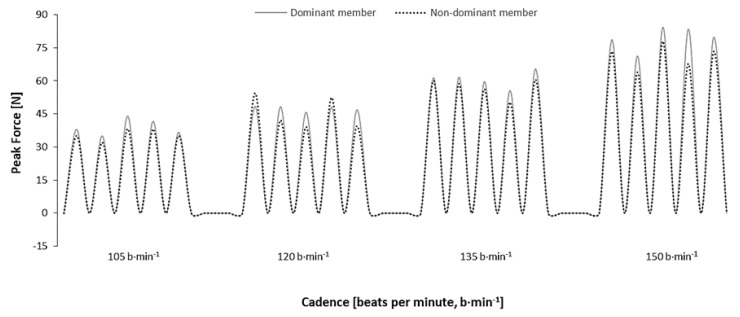
Example of the peak propulsive force between the dominant (solid line) and nondominant limbs (dashed line) during horizontal adduction (HA) in incremental protocol.

Figure 2 depicts a typical force curve between the dominant and nondominant limbs’ force in HA, during the incremental protocol. The overall trend was to an increase in the absolute propulsive peak force from slower to faster cadences.

Higher values were found in the HA isometric peak force of the dominant limb on dry land (75.21 ± 34.10 N) when compared with the propulsive peak force of the dominant limb (PF_D_) acquired in the water condition for HA (Table 1).

Table 2 shows the rate of force production for the dominant limb (RateF_D_) during water incremental protocol. The trend was to find higher values in the RateF_D_ during the cadences increment, from ~45 % (105 b·min^−1^) to ~66% (150 b·min^−1^) in HA and from ~38% (105 b·min^−1^) to ~68% (150 b·min^−1^) for RH_add_. Significant differences were found for HA_105–120_ (*p* = 0.02, *d* = 0.33), HA_105–135_ (*p* < 0.01, *d* = 0.73), HA_105–150_ (*p* < 0.01, *d* = 1.12), HA_120–135_ (*p* = 0.04, *d* = 0.39), and HA_120–150_ (*p* < 0.01, *d* = 0.76). There were also meaningful differences in RH_105–135_ (*p* < 0.01, *d* = 1.01), RH_105–150_ (*p* < 0.01, *d* = 1.43), and RH_120–150_ (*p* < 0.01, *d* = 0.99). A large ES was observed between the cadence 105–150 b·min^−1^ for both exercises. No differences were found for HA_135–150_, RH_105–120_, RH_120–135_, and RH_135–150_. However, RH_120–135_ showed a value close to the significance and a medium ES (*p* = 0.06, *d* = 0.52).

Table 3 presents the rate of force variation between cadences in HA and RH_add_. Lower cadences present a higher percentage in both exercises. In HA, the higher percentage occurred between the cadence 120–135 b·min^−1^, while, in RH_add_, it seems that 105–120 b·min^−1^ promoted the highest variation. Nevertheless, the cadence 135–150 b·min^−1^ presented the lowest percentage for the two exercises.

Table 4 reports the symmetric index (SI, %) for HA and RH_add_. The symmetric motion was found for HA at cadence 105, 120, and 135 b·min^−1^. However, RH_add_ elicited an asymmetric motion during the complete incremental protocol, and it seems that musical cadence of 135 b·min^−1^ promotes a value close to the symmetric motion.

## 4. Discussion

The aim of the present study was to analyze the rate of force production and the rate of force variation and to assess the symmetry during the exertion of the horizontal adduction and rocking horse observed in an incremental protocol. The main findings were that increments in cadences created an increase in the propulsive force, reaching ~68% of dry-land maximal force. Moreover, the cadence of 135 b·min^−1^ elicited a more symmetric action in both water fitness exercises.

Our results showed that both exercises elicited close to 50 N at the fastest musical cadence (150 b·min^−1^). Becker and Havriluk [8] conducted a study in swimmers during the horizontal adduction exercise at maximum velocity and reported values near 76 N and 80 N for the right and left hand, respectively. Differences in results may be explained by limit of exertion. In this sense, our study imposed a limit on the segmental frequency by the musical cadence, which mean that exertion may not have been led up to the maximum velocity of the subjects.

There was a trend to obtain higher values of the propulsive forces through the increase of the musical cadence. The increase in music is expected to induce an increase in limbs’ velocity and, as a consequence, in drag force [22]. Previous studies using CFD verified the maximum value of drag force when the hand adopted an angle attack of approximately 90˚, near perpendicular to the flow [23]. This was the case in this study, where the maintenance of the hand at 90˚ promoted higher forces in HA and RH_add_. Despite some differences already reported between HA and RH_add_ at lower cadences [24], both exercises seem, at this stage, to induce a similar strength exertion.

The American College of Sports Medicine [25] guidelines recommend 8–12 repetitions per set with ~60%–80% of the one repetition maximum (1-RM) in 2–3 days·week^−1^ to improve muscular strength and mass on dry-land programs. Standard guidelines for water fitness programs are set by the Aquatic Exercise Association [26], considering the American College of Sports Medicine’s guidelines.

To the best of our knowledge, this study is the first clarifying the strength level that is applied in water by considering dry-land strength assessment. The RateF_D_ reached from ~38% to 68% of the IsometricF_D_, according to exercise and the musical cadence. When analyzing both exercises, it seems that RF_D_ for HA was higher compared to RH_add_, except at the 150 b·min^−1^. This may be explained by the variability of the limbs’ range of motion when a static (e.g., HA) or an imbalance position (e.g., RH_add_) is adopted. Barbosa et al. [27] demonstrated that the range of motion was maintained while performing the rocking horse at higher cadences. However, the multiple hops can lead to an instability, leading to lower values in lower cadences when compared with the HA. This suggests, as happens in the physiologic domain [16], that an exercise using the upper and lower limbs does not promote higher exertion than the exercise that used only upper-limb motions. Moreover, we were interested in examining how the rate of force increase develops along an incremental protocol_._ The results showed an increased in the cadence 105–120 b·min^−1^ and also in 120–135 b·min^−1^ for HA and RH_add_. Interestingly, the cadence 135–150 b·min^−1^ promoted the lowest rate in both exercises, eliciting the higher exertion between them. The relationship between strength and propulsive forces still remains unclear [28]. These values present the first approach to quantify the force generation that the water environment allows a subject to perform during the water fitness exercises. The traditional guidelines need an adjustment for water fitness programs. This means adding the musical cadence near to the load of intensity in the guidelines chart to promote an ideal condition of strength development. It is important to mention that this study was conducted with young subjects, and a hypothetical increase in cadence in adults and elderly people can lead to a decrease in optimal control to follow the rhythm.

The lateralization phenomenon that characterizes symmetry can be established early in human life [29]. Within this rational, the body side choice plays an important role when asymmetries arise in any type of motion. Many factors can explain this phenomenon, as reported by Sanders, Thow, and Fairweather [30]: (i) bilateral imbalances; (ii) anteroposterior imbalances; and (iii) deficits in strength. Within the aquatic activities, the action of the upper limbs was considered to be mainly responsible for locomotion [14]. Likewise, the adduction and abduction of upper limbs can reach a higher range of motion [31], especially when four limbs are in action. The current study aimed at quantifying the asymmetries imposed by the incremental protocol. The RH_add_ showed a trend for a more asymmetric pattern than HA for all musical cadences. Requiring an alternative segmental action, RA_add_ claim some optimal level of coordination between upper and lower limbs. When analyzing the kinematics of “side kick”, Oliveira et al. [32] reported a reduction in the vertical displacement of the center of mass to follow the higher musical cadences. Exercises that require an imbalanced position with jumping at lower cadences will necessarily require more time to return to the ground contact. This may be the main reason RH_add_ reached only the value near to the symmetric motion at 135 b·min^−1^. Nonetheless, both exercises elicited an asymmetric motion at 150 b·min^−1^. Probably, three factors can helps explain such phenomenon: (i) the motor control decrease during the action of limbs to reach the cadence; (ii) the exhaustion/fatigue by the higher intensity of exertion; and (iii) the hypothetical decrease in elbow range of motion. Indeed, our results suggest that a musical cadence of 135 b·min^−1^ is appropriate to minimize the asymmetries during the execution of water fitness exercises while working strength.

Some additional limitations can be addressed in our research: (i) the uncontrolled effect of range of motion (i.e., kinematic analysis); and (ii) the use of young, healthy adults as subjects in one activity that is primarily chosen by other cohorts (e.g., older adults or those with disabilities).

## 5. Conclusions

Increasing musical cadence promotes an increase in force production by upper limbs in water fitness exercises. The musical cadence of 135 b·min^−1^ seems to elicit the optimal rate of force without compromising the motion pattern. Moreover, the horizontal adduction elicited a symmetric motion in most of the cadences and can be considered the most suitable exercise to build up strength.

## Figures and Tables

**Table 1:** Descriptive statistic (Mean ± SD) of the propulsive peak force of dominant limb in two head-out water exercises at different cadences.

Variables		Cadence (b·min^−1^)		
	105	120	135	150
HA				
PropulsiveF_D_ (N)	31.45 ± 12.13	35.81 ± 13.04	41.93 ± 14.06	47.66 ± 14.42
RH_add_				
PropulsiveF_D_ (N)	25.67 ± 8.15	32.40 ± 10.39	40.57 ± 12.83	48.42 ± 14.68

b·min^−1^, beats per minute; HA, horizontal adduction; RH_add_, rocking horse adduction; PropulsiveF_D_, propulsive peak force of dominant limb.

**Table 2:** Descriptive statistic (Mean ± SD) of the rate force production in two head-out water exercises at different cadences.

Variables		Cadence (b·min^−1^)		
	105	120	135	150
HA				
RateF_D_ (%)	44.77 ± 17.46	50.98 ± 19.33 ^α,^*	59.03 ± 20.95 ^α,^**, ^β,^*	66.43 ± 20.47 ^α,^**, ^β,^*
RH_add_				
RateF_D_ (%)	37.75 ± 17.20	46.69 ± 18.07	56.59 ± 19.32 ^α,^**	67.90 ± 23.64 ^α,^**, ^β,^**

b·min^−1^, beats per minute; HA, horizontal adduction; RH_add_, rocking horse adduction; RateF_D_, rate of force production in dominant limb; * *p* ≤ 0.05; ** *p* ≤ 0.01; ^α^, different from 105 b·min^−1^; ^β^, different from 120 b·min^−1^.

**Table 3:** Rate of force variation in the incremental protocol (Mean ± SD).

Variables	Cadence (b·min^−1^)		
	105–120	120–135	135–150
HA			
ΔForce (%)	12.40 ± 10.30	13.92 ± 11.05	10.53 ± 18.81
RH_add_			
ΔForce (%)	19.07 ± 15.77	18.04 ± 17.15	14.43 ± 19.68

b·min^−1^, beats per minute; ΔForce, variation of force increases between cadences.

**Table 4:** Descriptive statistic (Mean ± SD) for the symmetric index (SI).

Cadence (b·min^−1^)	Variable	HA	RH_add_
		Mean ± SD	Mean ± SD
105	SI (%)	10.49 ± 8.25 ^(a)^	14.11 ± 10.77 ^(b)^
120	SI (%)	10.50 ± 7.80 ^(a)^	14.33 ± 10.38 ^(b)^
135	SI (%)	9.23 ± 5.20 ^(a)^	12.71 ± 8.13 ^(b)^
150	SI (%)	11.85 ± 7.01 ^(b)^	15.35 ± 11.03 ^(b)^

b·min^−1^, beats per minute; SD, standard deviation; SI, symmetric index; ^(a)^, symmetric motion; ^(b)^, asymmetric motion.
